# *Tropheryma whipplei* Endocarditis: Case Presentation and Review of the Literature

**DOI:** 10.1093/ofid/ofy330

**Published:** 2018-12-07

**Authors:** Michael McGee, Stephen Brienesse, Brian Chong, Alexander Levendel, Katy Lai

**Affiliations:** 1 John Hunter Hospital, New Lambton, NSW, Australia; 2 University of Newcastle, Newcastle, NSW, Australia; 3 Tamworth Rural Referral Hospital, Tamworth, NSW, Australia

**Keywords:** endocarditis, *Tropheryma whippeli*, Whipple’s disease

## Abstract

Whipple’s disease is a rare infective condition, classically presenting with gastrointestinal manifestations. It is increasingly recognized as an important cause of culture-negative endocarditis. We present a case of Whipple’s endocarditis presenting with heart failure. A literature review identified 44 publications documenting 169 patients with Whipple’s endocarditis. The average age was 57.1 years. There is a clear sex predominance, with 85% of cases being male. Presenting symptoms were primarily articular involvement (52%) and heart failure (41%). In the majority of cases, the diagnosis was made on examination of valvular tissue. Preexisting valvular abnormalities were reported in 21%. The aortic valve was most commonly involved, and multiple valves were involved in 64% and 23% of cases, respectively. Antibiotic therapy was widely varied and included a ceftriaxone, trimethoprim, and sulfamethoxazole combination. The average follow-up was 20 months, and mortality was approximately 24%. Physician awareness is paramount in the diagnosis and management of this rare condition.

Whipple’s disease is a rare infective condition, classically presenting with gastrointestinal manifestations, first described a little over a century ago. The global prevalence of Whipple’s disease is unclear; however, *Trophyrema whipplei* has been detected in the stools (1%–11%) and saliva (0.2%–3.5%) of healthy individuals, varying on geography [1]. The epidemiology of cardiac involvement is not known. The responsible organism—*Trophyrema whipplei*—was only cultured with reproducible success in 2000 [2]. With the advent of improved molecular techniques, it is increasingly recognized as an important cause of what has traditionally been referred to as culture-negative endocarditis [3]. As compared with other causes of endocarditis, Whipple’s is a cause of subacute or chronic endocarditis, or in more modern terms, a nonacute, native, community-acquired endocarditis [4].

The largest review in the literature consisted of 35 patients and was published over 15 years ago [3]. Presentations of Whipple’s endocarditis continue to be nonspecific and poorly characterized, with treatment frequently requiring valvular surgery due to late presentation or delayed diagnosis and requiring long-term antibiotics [5, 6]. Further, the length of efficacious antibiotic treatment and the risk of relapse remain unclear.

Conclusive diagnosis is dependent on obtaining tissue, often at the time of surgery. This is a result of the difficulty of diagnosis and the scarcity of cases. To date, there have been no randomized clinical trials to guide investigation and management, and treatment is based on limited observational studies and case series, leaving clinicians with a paucity of data [3].

Here, we present a case of Whipple’s endocarditis without associated signs or symptoms of gastrointestinal involvement, accompanied by a literature review of cardiac manifestations of Whipple’s disease.

## METHODS

### Search Strategy

**Figure 1.**
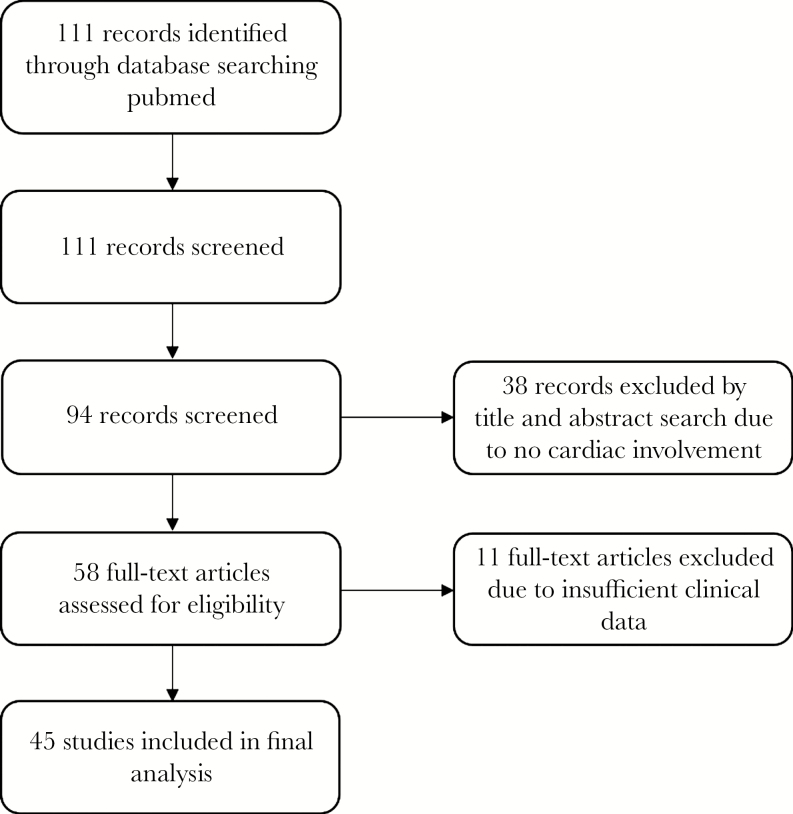
Consort diagram.

A systematic search of peer-reviewed articles was conducted using PubMed, as outlined in Figure 1. The search terms included “*Tropheryma*” and “endocarditis.” The search process was performed by the authors (M.M. and S.B.). This resulted in the identification of 111 peer-reviewed articles. Each article was screened by title and abstract and assessed for its relevance to the subject and included in the review as per the predefined inclusion criteria. Seventeen articles were excluded due to a lack of novel case description, and 38 were excluded due to no definite cardiac involvement. Each full-text article was then reviewed by the authors for inclusion in the final analysis. Eleven full-text articles were excluded for insufficient clinical data. Forty-five articles were included in the final analysis.

#### Inclusion Criteria

Articles were included only where patients had a confirmed diagnosis of Whipple’s disease by serological, valvular, or extracardiac tissue examination polymerase chain reaction (PCR), periodic acid–Schiff (PAS), or immunohistochemistry (IHC). Further, there needed to be evidence of endocarditis either by direct histologic examination or imaging, as per the Duke criteria of infective endocarditis (IE). Case reports, case series, and reviews with novel cases were included. English and non-English papers were included. Each case was assessed for demographics, comorbidities, clinical manifestations, underlying cardiac abnormalities, diagnostic information, antibiotic treatment, laboratory findings, and patient outcomes, including but not limited to surgery and death. Meta-analysis was not performed due to the variable and limited reporting of clinical data and outcomes measured.

### Case Information

A 57-year-old truck driver from rural New South Wales, Australia, complained of severely limiting exertional dyspnea, which was progressive over months. Premorbidly, he was independent and physically active. His medical comorbidities included a bicuspid aortic valve, squamous cell carcinoma with previous neck dissection, and radiotherapy, hypertension, gout, dyslipidemia, and prior Q fever infection 20 years earlier. Regular medications included atorvastatin, perindopril, and allopurinol. He had mild to moderate alcohol intake and was a former smoker. He was referred to a cardiologist for further investigation of his symptoms. There were no associated fevers or gastrointestinal symptoms.

A transthoracic echocardiogram revealed severe aortic regurgitation and a probable vegetation on the aortic valve. The patient was then admitted to the local hospital with a provisional diagnosis of infective endocarditis. The treating team commenced him on empirical antibiotic treatment including benzylpenicillin, flucloxacillin, and gentamicin, and he was subsequently referred to our institution for consideration of urgent aortic valve replacement.

During his preoperative workup, further assessment for endocarditis occurred. In total, 4 sets of blood cultures were negative (taken before antibiotic administration), and his white cell count and C-reactive protein (CRP) were found to be not elevated. He had a normocytic anemia with a hemoglobin of 11 g/L. Autoimmune screening was unremarkable. Antibiotics were stopped soon after his transfer to our institution, and he showed no signs of deterioration over 1 week. The patient proceeded to surgery. The intraoperative findings were that he had a bicuspid aortic valve and a vegetation on the left cusp with destruction of the leaflet. A mechanical prosthesis was implanted, a 25-mm ATS Medical (Minneapolis, Minnesota) metallic valve, and further testing was performed to identify the etiology of infective endocarditis. He had a complicated surgical recovery with delirium, acute kidney injury, fluid overload, and a large pleural effusion. He was treated for culture-negative endocarditis with 4 weeks of intravenous benzylpenicillin.

Microscopy of the aortic tissue showed no polymorphs and no organisms on Gram stain. There was no growth on routine culture. Unfortunately, histology was not requested on the sample. Serum antinuclear antibody, antiphospholipid antibody, *Legionella* serology, *Brucella* serology, *Bartonella* serology, and serum Q fever PCR were all negative. Ultimately, *T. whipplei* was detected on valve tissue by PCR and 16S rRNA gene sequencing. PCR on a postoperative pleural aspirate was also positive. The patient declined central nervous system (CNS) screening with a lumbar puncture.

Further history was obtained from the patient. He reported intermittent arthralgias, which was evaluated in the orthopedic clinic 12 months before admission, along with mono-articular knee arthritis. He also reported 25 kg of weight loss in the 6 months before his subsequent presentation.

He was commenced on combination trimethoprim and sulfamethoxazole therapy for 18 months. His recovery was otherwise uncomplicated, and he had regained 10 kg of weight at 3-month follow-up. He was alive and living independently at home 18 months after discharge from the hospital.

### Literature Review Results

The systematic search identified 169 patients (including the above patient) with Whipple’s endocarditis reported between 1999 and 2016. Previously reported cases were excluded in the final analysis. Results are summarized in Table 1 and Table 2. The average age (range) was 57.1 (33–80) years, and 144 (85%) were male.

**Table 1.** Sources From Which Cases Were Extracted

Author	Year	Journal	Country of Publication	Cases
Garcua-Alvarez [9]	2016	Medicine (Baltimore)	Spain	17
Gruber [17]	2015	Medical Case Reports	Switzerland	1
Damaraju [18]	2015	NEJM	Canada	1
Jos [5]	2015	BMC Research Notes	France	1
Emonet [19]	2015	International Journal of Infectious Diseases	Switzerland	1
Borne [20]	2015	The American Journal of Medicine	USA	2
Alozie [6]	2015	BMC Infectious Disease	Germany	2
Herrmann [21]	2014	The Annals of Thoracic Surgery	Germany	1
Loughran [22]	2014	Journal of Clinical Pathology	UK	1
Fenollar [23]	2013	International Journal of Antimicrobial Agents	France	2
Weisman [24]	2013	Canadian Journal of Infectious Diseases and Medical Microbiology	Canada	1
Fenollar [10]	2013	Emerging Infectious Diseases	France	28
Algin [25]	2012	BMC Research Notes	Netherlands	1
Love [26]	2012	Interactive CardioVascular and Thoracic Surgery	UK	1
Geißdörfer [27]	2012	Journal of Clinical Microbiology	Germany	14
Agard [28]	2012	Scandinavian Journal of Rheumatology	France	2
Chan [29]	2011	International Journal of Infectious Diseases	Canada	1
Whistance [8]	2011	Journal of Heart Valve Disease	UK	1
Ansemant [30]	2010	Joint Bone Spine	France	1
Escher [31]	2010	Clinical Microbiology and Infection	Switzerland	4
Besnard [32]	2010	American Journal of Medicine	France	4
Lagier [33]	2010	Medicine	France	16
Le Scanff [34]	2008	La Revue de Medecine Interne	France	2
Voldstedlund [35]	2008	APMIS: Acta Pathologica, Microbiologica, et Immunologica Scandinavica	Denmark	2
Williams [36]	2007	New England Journal of Medicine	UK	1
Kolek [37]	2007	Klin Mikrobiol Infekc Lek	Czech Republic	1
Marin [38]	2007	Journal of Clinical Microbiology	Spain	1
West [39]	2005	Journal of the Royal Society of Medicine	UK	1
Aiouaz [40]	2005	La Revue de Medicine Interne	France	1
Saba [41]	2005	Presse Medicale	France	1
Lepidi [42]	2004	Journal of Infectious Diseases	France	5
Dreier [43]	2004	Journal of Clinical Microbiology	Germany	1
Marrakchi [44]	2004	La Tunisie Medicale	France	1
Richardson [45]	2003	The Journal of Infection	Canada	2
Grijalva [46]	2003	Heart (British Cardiac Society)	Czech Republic	1
Bosshard [47]	2003	Clinical Infectious Disease	Switzerland	1
Geißdörfer [48]	2001	Infection	Germany	1
Geißdörfer [49]	2001	European Journal of Clinical Microbiology and Infectious Diseases	Germany	1
Ferrari [50]	2001	Revista do Instito de Medicina Tropical de Sao Paulo	Brazil	3
Charniot [51]	2001	Archives des Malidies du Coeur et Des Vaisseaux	Tunisia	2
Fenollar [3]	2001	Clinical Infectious Disease	France	35
Smith [52]	2000	Annals of Internal Medicine	USA	1
Wolfert [53]	1999	Southern Medical Journal	USA	1
Pron [54]	1999	European Journal of Clinical Microbiology and Infectious Diseases	France	1

**Table 2.** Summary of Literature Review

Characteristic	Number	Percentage
Number of patients	169	
Age of patients, y	57.1	
Male	144	85
Known valvular abnormality	36	21
Valve affected		
Aortic	108	64
Mitral	33	20
Tricuspid	6	4
Multiple valves	40	23
Presenting symptoms		
Fever	36	21
Arthralgia	87	52
Weight loss	42	25
Heart failure	70	41
Central nervous system	26	17
Outcomes		
Valvular surgery	125	74
Death	29	24

Cases were assessed for comorbidities or other predisposing risk factors but were not uniformly reported. Nineteen patients (11%) were reported to have some form of immunosuppression (immosuppresive medications such as steroids or autoimmune disease, predominately seronegative arthritis).

### Symptomatology

Overall, the preceding symptoms were largely not reported; however, 22 patients documented time from first symptoms to diagnosis, with an average of 21 months. Presenting symptoms were reported as articular involvement in 87 (52%), heart failure in 70 (41%), constitutional or systemic symptoms such as weight loss in 42 (25%), gastrointestinal symptoms in 34 (21%), fever in 36 (21%), and CNS manifestation such as emboli in 26 (17%). Several case reports documented diagnoses of psoriasis, seronegative, gout or rheumatoid arthritis which had been made more than 12 months prior to Whipple’s diagnosis and resolved with antibiotic treatment.

Of note, anemia was the most reported finding, in 67 (40%) of cases. Other laboratory findings were scantly reported, with CRPs ranging from 2.3 to 137 mg/L and B-type natriuretic peptide (BNPs) of up to 2536 ng/L in patients who had heart failure.

### Diagnosis

A diagnosis of definite Whipple’s endocarditis by direct examination of the valve was reported in 36 articles, accounting for 156 patients. Of these, 51% reported positive IHC, 72% reported positive PCR, and 39% reported positive PAS on valve tissue. A diagnosis of possible endocarditis (vegetations on valve imaging, culture-negative, Whipple’s diagnosis, and no other organism identified) was reported in 7 articles, accounting for 7 cases. In these patients, 85% had positive PCR on any tissue (duodenal, stool, salvia, or CNS), and 57% had positive PAS on a tissue specimen (duodenal biopsy or lymph node). In these 6 patients, there was insufficient information to establish a diagnostic method. 

### Valvular Disease and Involvement

Preexisting valvular abnormalities were reported in 36 (21%) patients, with aortic abnormalities being the most common (13, 8%). Three cases involved patients with previous valve replacements (2%). The aortic valve was the most common valve, involved in 108 (43%), followed by mitral (33, 20%) and tricuspid (6, 3%). Forty (23%) cases reported involvement of multiple valves, which were the aortic valve in combination with either the mitral or tricuspid valve. One patient had pericardial involvement in addition to endocarditis.

Unfortunately, vegetation size and appearance were rarely reported. Of those publications that did report vegetation size, the minimum vegetation size was 5 mm and the maximum was 33 mm.

### Treatment

Antimicrobial treatment initially consisted predominantly of intravenous ceftriaxone in 39 (23%), followed by trimethoprim/sulfamethoxazole in 67 (39%), or doxycycline ± a second agent in 34 (20%). The average treatment length (range) was 17 months (12 months to indefinite).

### Surgical and Mortality Outcomes

Of the 169 patients, 125 (74%) proceeded to surgery for valve replacement. The indication for surgery was not routinely reported but is assumed to be due to severe regurgitation. Twenty-four articles, accounting for 122 patients, reported at least 1 month of follow-up for at least 1 patient. The average length of follow-up was 20 months. Mortality was reported in 29 (24%) of these patients.

## DISCUSSION

Our case, in conjunction with a review of the literature, highlights several features of Whipple’s endocarditis that are unusual compared with the classical teaching about Whipple’s disease and indeed other causes of bacterial endocarditis. Arthralgia is the most common presenting complaint, which in our case was documented more than a year before the presentation that resulted in the diagnosis. Several cases, including ours, reveal that the arthralgia is often misdiagnosed in both classical and cardiac Whipple’s disease [7, 8].

Garcia-Alvarez et al. described a large contemporary case series including 17 cases recruited by Spanish registry data for infective endocarditis [9]. All cases were culture negative, with a positive diagnosis made by PCR of valvular tissue in 16 cases. In 1 case, in whom cardiac valve surgery was not performed, PCR was positive on intestinal biopsy, cerebrospinal fluid, and synovial fluid. Similarly, cases with extravalvular symptomatology demonstrated positive PCR testing. PCR testing was positive in 1 case with classical gastrointestinal symptoms on intestinal biopsy and 1 case with chronic pericarditis in the pericardial fluid. PAS staining was positive in 5 of 6 cases tested.

Fenollar and his co-authors have contributed greatly to our understanding of *T. whipplei* endocarditis, publishing some 65 novel cases and the largest case series with 28 cases, with patients recruited from their hospital, a self-described referral center for patients with *T. whipplei* in France [10]. Positive diagnosis was made on PCR testing of the affected valve in 27 cases.

For reasons that remain unclear, there is a strong sex predisposition among males for Whipple’s endocarditis. Similarly, Whipple’s disease has been described to be more common in white males of European decent, suggesting genetic predisposition [11], but this has not been conclusively proven.

Whipple’s disease has been regarded as a great mimicker of various diseases. It is generally considered in cases involving the 4 cardinal symptoms: arthralgias, diarrhea, abdominal discomfort, and weight loss. The medical presentation that resulted in the diagnosis of endocarditis for our patient was that of heart failure. This was the case for approximately 40% of patients, who similarly presented with heart failure. This is twice the rate compared with previous reviews. This may be explained by the high rate of surgical management of valvular disease. Further, surgical intervention allowed for conclusive diagnosis of a condition that may not have been otherwise considered. Diagnosis has been shown to be difficult in patients who do not have gastrointestinal symptoms [12]. Conversely, previous reviews were consistent with our findings that fever was a presenting feature in only one-quarter of patients. Although Whipple’s disease is rare and presents with multiorgan involvement, to the best of our knowledge, cardiac manifestations are seldom considered in diagnostic evaluation in contemporary series. As previously reported, there is a high prevalence of structurally normal valves compared with other bacterial infection series. Unlike other native valve infections, such as *Bartonella*, no predisposing factors have been identified.

Endocarditis due to *T. whipplei* remains a challenging diagnosis for a variety of reasons. The first is that patients do not present with classic features of endocarditis—they often have no fever, peripheral stigmata, or inflammatory response—and are hence unlikely to meet the Duke criteria for endocarditis [3]. Second, *T. whipplei* is only cultured in specialized laboratories, and hence would not be detected via routine blood and tissue culture [12]. Third, although PCR is useful in the diagnosis of Whipple’s disease, its yield varies depending on specimen type. For example, in a French case series, PCR of peripheral blood was positive in only 5 of 16 (31.2%) patients with Whipple’s endocarditis confirmed on histology or PCR of valve tissue [10]. On the other hand, a positive PCR result from a nonsterile site is not specific, as *T. whipplei* DNA has been detected in saliva and gut biopsy specimens of patients without clinical evidence of disease [13–15].

Finally, the role of serological tests in the diagnosis of Whipple’s disease is uncertain because patients with active Whipple’s disease may paradoxically have a lower immune response to *T. whipplei* compared with healthy carriers [16]. Due to the above factors, the diagnosis of Whipple’s endocarditis is often missed until the patient proceeds to valve replacement, at which point the opportunity for early treatment has been missed.

The optimal treatment of these patients in the long term remains uncertain. Antibiotic treatment recommendations are based on data from small observational studies to date, which consist of an initial phase with intravenous ceftrixaxone or penicillin, followed by a prolonged maintenance phase of cotrimoxazole and sulfamethoxazole for at least 12 months, or alternatively, doxycycline with or without hydroxycholoroquine.

Rates of death with Whipple’s endocarditis are significant and comparable to other causes of infective endocarditis in the modern era, at approximately 30% [4]. This could be due to the chronic nature of the disease, which allows time for cardiac adaptation.

The greatest challenge for management of Whipple’s endocarditis remains the diagnosis, further compounded by its rarity, despite advances in molecular techniques such as PCR. These patients are often afebrile, culture-negative, and have paradoxically low inflammatory markers despite signs of chronic inflammation including anemia and hypoalbuminemia. Consequently, diagnosis is often made postsurgery when the valve appears consistent with endocarditis and is sent for further examination. The optimal treatment of these patients in the long term remains unclear. Clinicians should consider the diagnosis of Whipple’s endocarditis in culture-negative endocarditis, especially if there is a history of any form of arthralgia or weight loss with low or normal inflammatory markers. Clinician awareness remains the cornerstone for identification and initiation of appropriate treatment.
